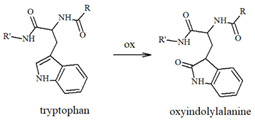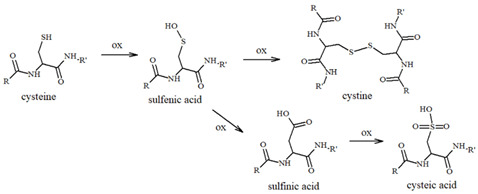# A Focus on the Link Between Metal Dyshomeostasis, Norepinephrine, and Protein Aggregation

**DOI:** 10.3390/antiox14030347

**Published:** 2025-03-15

**Authors:** Chiara Bacchella, Andrea Capucciati, Enrico Monzani

**Affiliations:** 1 Dipartimento di Chimica, Università di Pavia, Via Taramelli 12, 27100 Pavia, Italy; enrico.monzani@unipv.it; 2 Fondazione Grigioni per il Morbo di Parkinson, Via Gianfranco Zuretti 35, 20125 Milano, Italy

**Keywords:** norepinephrine, oxidative stress, redox active metals, post-translational protein modification, protein aggregation, neuromelanin

## Abstract

Neurodegenerative disorders are one of the main public health problems worldwide and, for this reason, they have attracted the attention of several researchers who aim to better understand the molecular processes linked to the etiology of these disorders, including Alzheimer’s and Parkinson’s diseases. In this review, we describe both the beneficial and toxic effect of norepinephrine (NE) and its connected ROS/metal-mediated pathways, which end in neuromelanin (NM) formation and protein aggregation. In particular, we emphasize the importance of stabilizing the delicate homeostatic balance that regulates (*i*) the metal/ROS-promoted oxidation of catecholamines, as NE, and (*ii*) the generation of oxidative by-products capable of covalently and non-covalently modifying neuroproteins, thus altering their stability and their oligomerization; these processes may end in (*iii*) the incorporation of protein conjugates into vesicles, which then evolve into neuromelanin (NM) organelles. In general, we aim to provide an up-to-date overview of the challenges and controversies emerging from the current literature to delineate a direction for future research.

## 1. Norepinephrine Metabolic Alterations and Their Implication in Neurodegeneration

Neurodegenerative disorders are progressive fatal diseases that affect the central neural system (CNS); the most common form are Alzheimer’s and Parkinson’s diseases. The early signs of neurodegeneration mainly occur in the noradrenergic system, which is the principal pivotal neuromodulator in the brain, and consist of the formation of protein aggregates piled up at axons and dendrites, thus hampering the normal release of neurotransmitters containing vesicles [1,2]. *Locus coeruleus* (LC) is one of the main brain regions affected by neurodegenerative decline in AD and PD, likely a result of its high propensity for the accumulation of early protein aggregates.

One of the principal stimuli for protein aggregate deposition is the presence of heavy metal ions in their oxidized state; indeed, amyloid plaques, tau tangles and α-synuclein Lewy’s bodies contain high concentrations of copper, iron and zinc ions [3,4,5], where Cu and Fe ions exert increased toxicity due to their redox activity, which results in oxidative stress-mediated chemical damage in neurons. In particular, it is believed that, in physiological conditions, these metal ions mainly exist as reduced species because of the presence of consistent levels of reducing molecules, including glutathione (GSH) and ascorbic acid (AA) [6,7]; these reductants are fundamental to maintain the balance between the reducing pathways and the oxidative reactions promoted by the presence of superoxide and hydrogen peroxide. Unfortunately, this delicate equilibrium is totally lost when the availability of GSH and AA is reduced and the oxidative stress levels are increased, thus leading to the unspecific oxidation of biotargets, i.e., the generation of disulfide bridges and the further oligomerization of neuroproteins [8].

Small amounts of ROS are vital for the homeostatic control of the different functions associated with cell differentiation, proliferation, apoptosis and redox-sensitive signal transduction pathways [9,10]; on the other hand, the CNS contains the tissues most vulnerable to oxidative stress in the body due to its high oxygen consumption (approximately ten times the average needed by other tissues), which is necessary for high-energy signal transduction. Besides metal imbalance-mediated ROS generation, another source of oxidizing reactive species comes from the activation of the immune system. Indeed, neuroinflammation is a normal repair process promoted by the activated immune system when CNS injury occurs; however, when the damage repair fails, a chronic inflammation state prevails, thus resulting in a steady production of ROS by fibroblasts, lymphocytes and vascular endothelial cells [11,12].

The last source of ROS is the intrinsic reactivity of catecholamines, which form quinone derivatives; indeed, unlike other CNS-relevant biogenic amines, dopamine (DA) and norepinephrine (NE) are prone to undergoing non-enzymatic auto-oxidative reactions, which lead to hydrogen peroxide formation [13].

**Figure 1:**
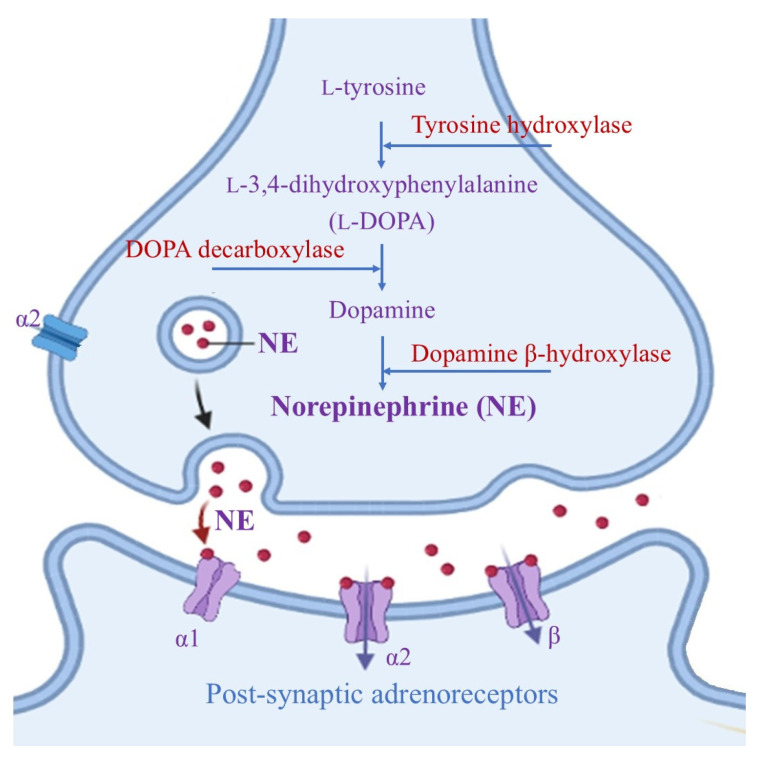
Biosynthesis of norepinephrine mediated by two key enzymes, dopamine β-hydroxylase and tyrosine hydroxylase (which catalyzes the rate-determining step in NE formation). After NE release at the synaptic level, it performs its neuromodulation activity via G-coupled α- and β- adrenoreceptors (where the α1 and β receptors are mainly present at post-synaptic sites and perform excitatory actions, while α2 receptors have an inhibitory effect on both pre- and post-synaptic areas).

LC is a small bilateral collection of neuromelanin-containing neurons that are usually immunochemically identified by the presence of dark pigments, as well as by the two key enzymes needed for norepinephrine (NE) biosynthesis (Figure 1) [14].

NE is a mono-aminergic neurotransmitter that is usually co-localized with neuronal proteins, including Aβ peptides, within storage vesicles and controls disparate functions in the modulation of cognition, sleep, emotion, learning, memory and stress response processes [15,16]; NE also influences blood flow, neuroinflammation and neuronal survival [17,18].

Alterations in catecholamine levels, including NE, may affect neurodegenerative decline by enhancing the formation of toxic DA and NE by-products, which are capable of contributing to organelle dysfunction, protein peroxidation and fibrillation [19]. For example, mitochondrial damage, protein (including α-synuclein, hyperphosphorylated tau, amyloid precursor protein (APP), and Aβ) [20,21] aggregation, and the loss of autophagy-lysosomal functions are detected after 3,4-dihydroxyphenylglycolaldeyde (DOPEGAL) administration as a result of increased free radical production [19,22].

On the other hand, the physiological functions of NE remain controversial; in AD, a significant decrease (approximately 31% and up to 50% upon two years from AD onset [23]) in NE concentrations was detected in the mid-temporal cortices. Epidemiological studies have suggested that the presence of polymorphisms in the dopamine β-hydroxylase gene, which controls NE availability in brain, results in a two-fold increased risk of developing AD in the subject [24].

**Figure 2:**
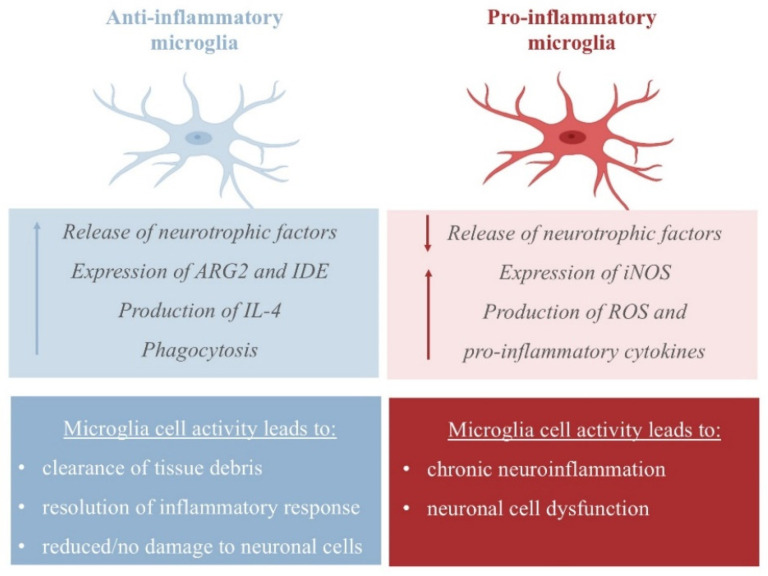
Pro- and anti-inflammatory activity of microglia. The pro-inflammatory state is characterized by the release of inflammatory cytokines and nitric oxide (NO) via the upregulation of inducible NO synthase 2 (iNOS/NOS2); the anti-inflammatory response is achieved by the release of anti-inflammatory cytokines, including IL-4 and IL-13, and the upregulation of arginase 2 (ARG2) and insulin-degrading enzyme (IDE) [30].

The neuroprotective activity of NE emerges from its ability to increase the expression of CCL2 chemokines by astrocytes, which have been found to protect neurons from injury [25], and its ability to inhibit dopaminergic neuron apoptosis [26]. In APP23 transgenic mice, NE represses microglial transcription pro-inflammatory genes, which are required for microglial migration and cytokine and chemokine release, and favors the transcription of mRNA-coding amyloid-β-degrading enzymes (such as the metallopeptidase neprilysin, NEP), thus assisting in plaque degradation [27,28]. Additionally, NE protects cortical neurons from a proinflammatory response to protein deposition by reducing the expression of nitric oxide synthase-2 (NOS2), thus managing free reactive species in the cortex (Figure 2) [29].

In vitro and in vivo studies have shown that the administration of NE and/or the promotion of NE biosynthesis confer long-term protection to the dopaminergic system: indeed, NE seems to exert a neuroprotective effect on oxidative stress, scavenging hydroxyl radicals and thus preventing neuromembrane lipid peroxidation [31]. Moreover, NE may upregulate the expression of γ-glutamylcysteine ligase as well as downregulate the glutathione peroxidase gene, resulting in an increased availability of glutathione, thus reducing metal ions and exerting a neuroprotective effect [32]. On the contrary, previous studies have shown that the reduced form of copper ions is able to promote hydroxyl radical generation in the presence of catecholamines, including NE, thus decreasing the levels of intracellular GSH and the SOD enzyme; this results in oxidative damage, the generation of toxic DNA adducts, and neuronal death [33,34,35].

Besides copper ions, iron ions are also the active in the promotion of aberrant oxidation of NE in the presence of molecular oxygen; on the other hand, in vitro studies have suggested that iron(II) chelation by NE prevents ROS generation by partially suppressing the Fenton reaction [36]. At the same time, the presence of high ATP concentrations in LC neurons was demonstrated to be essential in stabilizing iron(III)/NE complexes to give a moderately reactive ternary species, thus slowing down NE oxidation and the iron-catalyzed Fenton/Haber–Weiss pathways [37].

A related pathway that could contribute to LC degeneration is the formation of neuromelanins (NMs), which are insoluble inclusions accumulated in LC and in *substantia nigra* (SN) pigmented neurons during brain aging [38]; indeed, the high vulnerability of noradrenergic neurons arises from the aberrant oxidation, especially when catalyzed by metals, of NE to *o*-quinone products. These species are highly reactive toward *L*-cysteine (circulating or inside proteins) and glutathione, thus leading to neurotoxic conjugates. It was proposed that NM formation may be initiated by the neuronal accumulation of β-structured protein aggregates, which act as protein growth nuclei [39,40,41,42,43]. For example, the co-localization of the misfolded α-synuclein protein and NMs was suggested, reaching particularly high levels of fibrillar protein when associated with the NM lipid components [44]. These fibrillar seeds are then responsible for consecutive reactions with catecholamine metabolites (including NE derivatives), giving highly polymerized melanin–protein conjugates; redox metal ions, particularly iron(III), actively promote these processes, resulting in the generation of non-structurally organized and insoluble components containing high levels of cross-β proteins and reactive metal ions, which are finally accumulated within the autophagic vacuoles. These highly damaged inclusions become undegradable by the proteasome system and are therefore piled up in neurons [45].

A deeper analysis of the contradictory functions of norepinephrine as well as its metal/ROS-mediated biological pathways is provided in the next sections.

## 2. Protein Aggregation: How Metals and Norepinephrine Affect Protein Fibrillation

The fibrillation of proteins is associated with the most common aging-related human diseases, such as Alzheimer’s, Parkinson’s, prion diseases, and type 2 diabetes [46,47,48]. Metal imbalances, particularly imbalances of copper, iron and zinc ions, deeply affect the stability and functionality of different biological components, such as proteins and, in particular, enzymes. A vicious cycle between metal dysregulation, oxidative stress and protein aggregation is associated with neurodegenerative decline, ending in generalized inflammation, mitochondrial dysfunction and cell death by apoptotic and/or necrotic mechanisms.

In this context, neuronal vulnerability towards oxidative stress is enhanced by the presence of excess cytosolic catecholamines, which are not included in storage vesicles and are exposed to uncontrolled clearance mechanisms [49,50]. The metabolism of these redox active molecules easily generates significant amounts of ROS and the presence of metal ions abruptly accelerates the conversion of catecholamines into quinone species [49,51,52]. These oxidative products can react with nucleophilic side groups of peptides and proteins, such as the polar groups of cysteine, histidine and lysine residues, modulating their stability and conformational features; this results in two typical pathways: protein fibrillation, which is the topic of this section, and the incorporation of the conjugates into lysosomal vesicles that turns into neuromelanin organelles (described in Chapter 3) [42].

### 2.1. NE and Amyloid-β Peptides in AD

**Figure 3:**
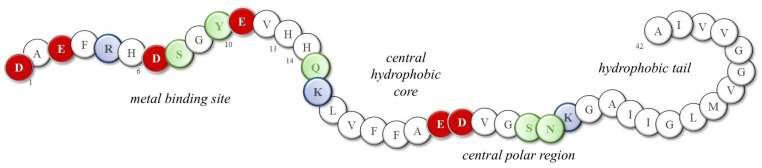
Sequence of the 42-residue Aβ peptide (Aβ(1–42)). Red: negative charge residues; Blue: positive charge residues; Green: polar; White: neutral and non-polar residues.

Although the toxicity of dopamine and its involvement in protein misfolding have long been recognized, little progress has been made in understanding which structural and functional modifications may result from the presence of norepinephrine and, possibly, excessive metal ions. The presence of extracellular senile plaques and intracellular neurofibrillary tangles (NFTs), mainly constituted by amyloid-β (Aβ) peptides and tau proteins, respectively, is the main histological evidence of Alzheimer’s disease [53,54,55]; the most abundant Aβ isoforms terminating either at amino acid 40 or at amino acid 42 (Aβ(1–40) and Aβ(1–42), respectively) are produced from the amyloid precursor protein through proteolytic cleavage and then released into the cortex and cerebrospinal fluid (CSF). In physiological conditions, Aβ monomers assume a highly disordered structure and their amphipathic nature promotes their interaction with both polar/charged and hydrophobic cellular components, including metal ions, neurotransmitters, phospholipids, etc. Indeed, Aβ peptides show the *N*-terminal hydrophilic segment 1–16, which is able to coordinate metal ions owing to the presence of three histidines, one tyrosine and four negatively charged residues (Figure 3). In fact, the high-affinity binding sites of copper(II) and zinc(II) ions (K_d_ values are approximately 1–50 nM and 0.1–1 µM, respectively) are provided by the imidazole side chains of His6, His13, and His14, as well as the *N*-terminal amine [56,57,58]; meanwhile, the coordination sphere of iron(II) ions possibly combines histidines with negatively charged Asp/Glu amino acids and/or Tyr residue [59,60,61]. Although some of the literature suggests that the formation of metal-Aβ species is a protecting event, reducing free metal levels and the related oxidative damage [62,63], it also worth noting that the coordination of these Aβ peptides to metal ions often activates their redox cycling, resulting in a higher release of reactive oxygen species (ROS) that target several biological components, including the peptides themselves [64,65,66,67,68].

#### 2.1.1. Oxidative Modifications Undergone by the Aβ Peptide

Our group has extensively characterized the oxidative products of Aβ during metal-catalyzed oxidation [69,70,71,72,73] and we have primarily identified His and Tyr residues as the main targets of ROS, although Phe and Met have also been found in their oxidated forms. Histidines, in particular His13 and His14 [74,75], have been identified as very sensitive to oxidation, leading to their conversion to 2-oxohistidines (Table 1) after a reaction with hydroxyl radicals [76,77]. The insertion of an *O*-atom into the imidazole ring occurs via a radical mechanism in which redox-active metal ions, such as copper, coupled to a reducing substrate, such as a catecholamine, play a key role. Similarly, the presence of metal ions and hydrogen peroxide makes Tyr10 highly sensitive to oxidation, resulting in the generation of dityrosine (Table 1), which covalently links two peptide chains. It is therefore reasonable to assume that metal-mediated oxidative damage on the Aβ peptides deeply alters the likelihood of oligomerization occurring, then evolving into neurotoxic peptide assemblies.

#### 2.1.2. NE as a Modulator of Aβ Damage and Aggregation

In addition to the deleterious impact of metal ions on peptide fibrillation, the relationship between catecholamine toxicity and protein aggregation has also attracted the attention of several researchers. In fact, norepinephrine and other catechol derivatives are found to be active in the destabilization of Aβ protofibrils and, based on this observation, NE related-compounds has been also suggested as potential therapeutic compounds for the treatment of AD. Norepinephrine is a catecholamine neurotransmitter produced within the *Locus coeruleus* (LC) and is co-localized with Aβ inside storage vesicles before catecholaminergic cell stimulation [78]. Multiple studies have shown the ability of DA and NE to modulate the stability of Aβ oligomers and prevent fibril formation [79,80]. Zou et al. [81] suggested, through molecular dynamic simulations, that the β-structures typically observed in Aβ dimers are strongly suppressed by the presence of NE molecules and are replaced by more disordered coil-rich structures. In particular, two favorable regions corresponding to the central hydrophobic core (16–21) and a *C*-terminal hydrophobic site encompassing the residues 31–36 (two crucial regions for the Aβ aggregation [82]) have been found to be essential for NE/Aβ interaction. On the other hand, this work also revealed the importance of there being negatively charged residues in Aβ (Asp1 and Asp23) for *H*-bonding interactions with NE molecules, in addition to significant hydrophobic and aromatic stacking with Phe4, Tyr10, Phe19, and Phe20 (Figure 3). Since norepinephrine is mainly in its protonated form at the physiological pH, Zou’s research group reported that two distinct mechanisms are involved in the disruption of Aβ oligomers depending on the NE charge: protonated NE primarily interacts with the *N*-terminal domain of Aβ peptides, hindering the development of His6-Glu11 side-chain *H*-bonds and increasing the kink angle around Tyr10. On the other hand, deprotonated NE strongly interacts with the aromatic residues His6, His13, and Phe20 through hydrophobic and *π-π* stacking interactions, thus resulting in the destabilization of fibrils [83].

The amyloid inhibitor activities of catechol-containing compounds, including NE and derivatives, have attracted the attention of several researchers with the aim of identifying new therapeutic compounds that are able to contrast amyloid deposition [84]. However, the role of NE in the modulation of protein-misfolding-associated neuronal damage remains controversial because of the diverse functions of Aβ and NE in AD pathology. The complexity of NE-related pathways was realized in the vitro characterization performed by Liu et al. [85], in which thioflavin T fluorescence and kinetic analysis confirmed the anti-aggregation activity of NE; however, this also suggested that there are cytotoxic effects on neuroblastoma SH-SY5Y cells depending on the NE concentration. Regarding the destabilizing mechanisms provided by the presence of NE molecules as fibril intercalators, it has been observed that the interaction of the OH/*π* hydrogen bond [86] between the negatively charged *π* face of Tyr10 and the positively charged *H*-atom of NE is the mechanism responsible for the NE/Aβ destabilization, besides the weak binding with the SNK(26–28) segment that only occurs at high NE concentrations. At the same time, since the preferential interaction of Aβ with NE occurs in the *N*-terminal hydrophilic domain, which is also the region responsible for copper binding, competitive binding to the Aβ mechanism between NE and copper(II) may occur (also suggested by the close K_d_ values of NE/Aβ, 0.25 μM, and Cu^2+^/Aβ, 0.04 μM observed at the physiological pH) [85].

**Scheme 1:**
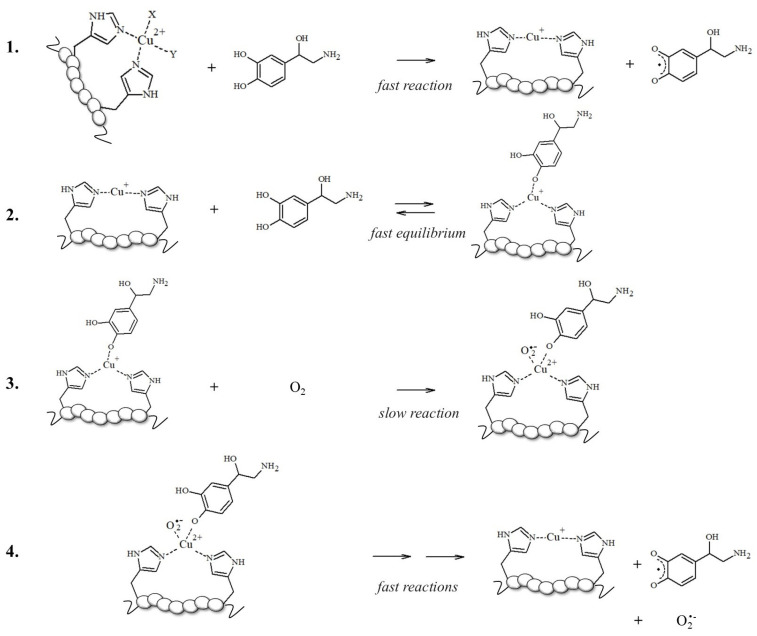
Schematized mechanism followed by [Cu-peptide] complexes for NE oxidation; reaction 3 is the rate-limiting step and gives to [Cu^2+^-peptide-NE/O_2_^•−^], a reactive and transient intermediate that is not isolable due to its fast decomposition [50].

To support this hypothesis, the authors found that the higher production of ROS assayed in aerobic solutions containing mixed Aβ, Cu^2+^, and NE reflects the competitive binding of NE vs. Cu^2+^ to Aβ; norepinephrine may limit the accessibility of copper to the *N*-terminal binding site, thus enhancing the free Cu^2+^ concentrations; in turn, this increases ROS formation via the Haber–Weiss and Fenton-like reactions, exacerbating the cytotoxicity in cells [87]. However, this increase in ·OH levels and the reduction in cell viability upon NE exposure could be also related to the Aβ-bound Cu redox cycling in which the electron transfer to oxidable substrates, such as NE, is facilitated when copper is bound to electron donors, such as imidazoles of His-containing peptides (Scheme 1) [50,70,71,88,89].

The effect of NE and its derivatives still needs to be elucidated, particularly when redox-active metal ions or other relevant biomolecules increase the complexity of the scenario. For example, it has been suggested that the oxidation of Met35 in Aβ can drive its amyloidogenic toxicity through radical mechanisms that are able to target and damage nearby biomolecules, such as lipids; on the contrary, this reaction seems to be inhibited by the presence of catecholamines and copper ions, which can further oxidize the sulfuranyl radical species to the stable methionine sulfoxide (Table 1), thus reducing Aβ-related toxicity [80,90].

**Scheme 2:**
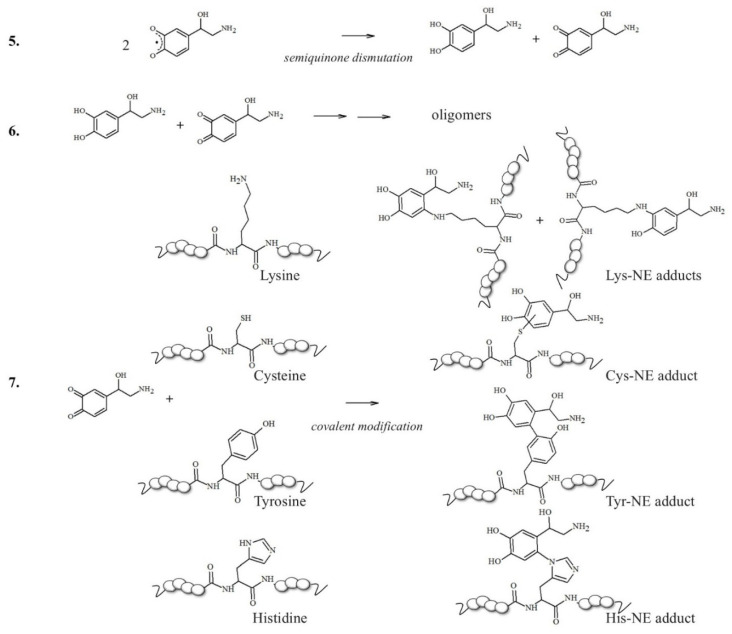
NE oxidation pathway leading to melanin formation and the covalent modification of sensible amino acid residues inside proteins/peptides.

The idea that a single post-translational modification may significantly contribute to the modulation of the toxicity of Aβ aggregates increases our interest in elucidating the effects (direct or indirect) of chemical alterations on the Aβ peptides. For example, two candidate sites for forming covalent linkages between catecholamines and Aβ peptides would be Lys16 and Lys28 residues (Scheme 2); in the brain, NE is normally maintained in its reduced state while it is stored in vesicles prior to exocytosis [91]; however, in oxidative stress conditions, NE can be easily converted into its *o*-quinone (NEoxide). Surprisingly, thanks to these oxidizing conditions, which are highly dangerous for neurons, the generated NE oxide can react with the two Lys (two key residues for intermolecular β-sheet structuring [92]) of Aβ via a Micheal addition reaction, and this single covalent modification seems to be effective in inhibiting amyloidogenic aggregation [93].

In addition to Met and Lys residues (Table 1), Tyr10 is of high interest because its phenolic nature makes it more prone to accepting electron transfer reactions that form a phenoxyl free radical [94]; the level of this species increases during neuronal degeneration due to the activity of metal-generated ROS, and it quickly evolves into dityrosine linkages (Table 1) between protein/peptide monomers. High levels of dityrosine crosslinks have been detected during aging and, in particular, the formation of dityrosine crosslinks between Tyr10 residues in Aβ peptides has been observed in the brain of AD subjects [95,96,97,98]. Allnutt et al. [99] suggested an increase in oligomerization and a decrease in the toxicity of these oligomeric species when Aβ aggregation occurs in the co-presence of norepinephrine. In particular, the physical characterization of Aβ aggregates has indicated that NE stabilizes oligomers in highly amorphous structures that lack dityrosine linkages (possibly due to the radical-scavenging ability of NE), which differs from the organized fibrillar species usually observed when Aβ is aggregated alone. Since NE-modulated Aβ fibrillation results in minor in vivo toxicity and an increased cell viability, a dual mechanism was hypothesized; in this, NE acts as a physical barrier to Tyr access, thus inhibiting Tyr-Tyr linkages and aggregation, and as an antioxidant and radical quencher. The Aβ(Tyr10)/NE interaction can be controlled both by covalent linkages and, as previously described in studies on binding free energy by Zou et al. [81], by non-covalent interactions through aromatic stacking between NE and the tyrosine residue. These coupled mechanisms significantly mitigate the toxicity of Aβ aggregates, also preventing the formation of the ROS normally released during the oxidation of amino acid residues. Therefore, in AD brains, where Aβ species are more extensively produced due to the altered processing of APP and the levels of neurotransmitters are reduced, the protective effect of catecholamines, including NE, is partially lost.

#### 2.1.3. NE Metabolites Linked to AD

**Figure 4:**
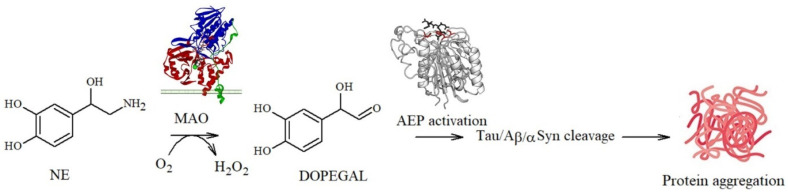
DOPEGAL-mediated pathway leading to tau/Aβ/αS fibrillation.

In addition, a delicate balance between the toxicity and protection exerted by NE and metabolites towards amyloidogenic processes has emerged: NE quinones and reactive semiquinones can also promote the depletion of glutathione, an important antioxidant molecule, and alter the functions of the macromolecules required for cell survival [100,101,102]. In addition, quinone derivatives can also activate asparagine endopeptidase (AEP), a lysosomal enzyme that specifically cleaves several proteins, including APP and tau, under conditions of acidosis. The cleavage products are known to be neurotoxic since they act as building blocks for Aβ deposition and tau propagation [22,103,104]. A similar pathway can be also activated by DOPEGAL, a metabolite generated via the oxidative deamination of NE by monoamine oxidase A (MAO-A) (Figure 4).

In 1999, Burke et al. [105] found significantly higher levels of DOPEGAL and MAO-A in AD neuronal cells when compared to the controls, suggesting a causal association with neuronal death during the progression of the disease. They also detected that, during the oxidative deamination of NE, the hydrogen peroxide released (Figure 4) [106] deeply altered the mitochondrial permeability, resulting in cell apoptosis [107]. Similarly, AEP activation also affects the production of propagation-prone tau species, since the resulting truncated forms at Asn255 and Asn368 lack microtubule assembly activity and are more susceptible to hyperphosphorylation and aggregation [22]; this also results in tau dislocation into the nucleus where the protein acts as a structural nucleic acid protein [108]. Moreover, as described for Aβ, tau protein aggregation can be also modulated by covalent modifications of its amino acid backbone: Kang et al. identified that DOPEGAL forms covalent adducts via Schiff base with Lys353 and that this single modification is able to accelerate tau fibrillation and hyperphosphorylation in cellular models, suggesting that it plays a potentially important role in promoting the disease [109].

### 2.2. NE and Tau Protein in AD

**Figure 5:**
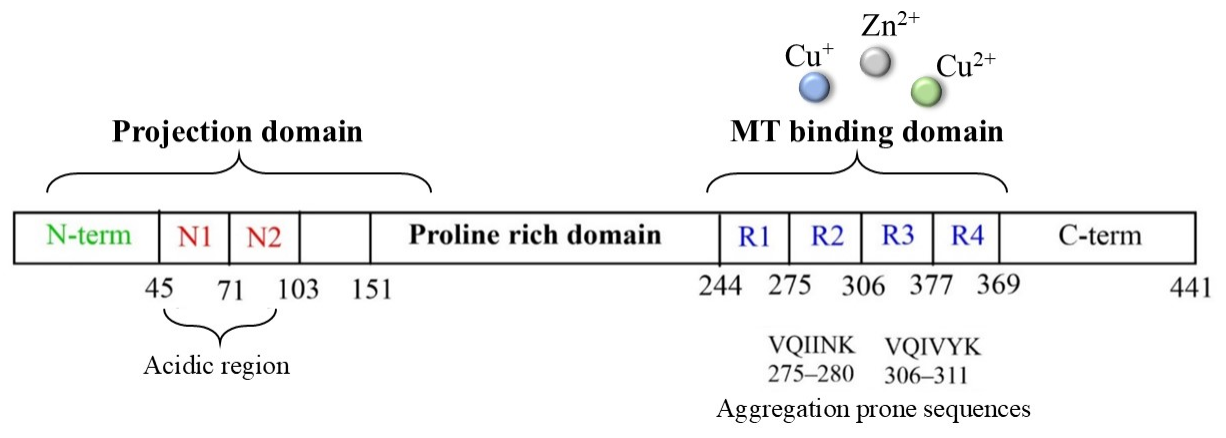
Scheme of tau protein and its microtubule-binding domain, which includes the four-repeat domain.

Tau protein is a microtubule-associated component that is widely distributed in the CNS and that, in normal conditions, is highly soluble and regulates neuronal structure and functions. In tauopathies, including AD, metal dyshomeostasis, dysregulated cellular metabolism and oxidative stress stimulate the post-translational modification of tau, which alters the hydrophobicity, folding and stability of the protein, thus triggering its aggregation. This loss of function, together with its conversion into a insoluble form, disrupts the weight-bearing structure of the neurons, leading to cellular death [110]. In this context, it is worth noting that the main targets of tau oxidative modifications are Cys291 and Cys322, two residues that are highly sensitive to the local redox changes and that are localized in the microtubule-binding region of the protein (particularly, in R2 and R3, respectively, Figure 5).

Cysteines can act as a fascinating molecular switch that is able to affect the conformation and functions of proteins through two interconnected mechanisms: (*i*) the presence of thiol groups assists in the generation of stable complexes between tau and some toxic transition metal ions, including copper, zinc and heme-iron, and the formation of complexes make the protein more prone to aggregation; (*ii*) cysteines can be used as the substrate of ROS/metal-catalyzed oxidative reactions to give *S-S* dimers (Table 1) and covalently modified tau monomers (Scheme 2). Dimerization via disulfide bonds seems to be promoted by the hemin-activated H_2_O_2_ or by the redox cycling of Cu ions; meanwhile, when catecholamines are present in the local medium, quinone derivatives quickly react with the nucleophile group of Cys through a Micheal addition reaction, resulting in the formation of high levels of covalent adducts [111,112]. On the other hand, some studies have revealed that mechanism *ii* may exert a protective effect regarding neurological decline due to the ability of catecholamines, in particular NE, to disrupt tau fibrillary aggregates and induce tau degradation [22,113]. Indeed, the addition of NE molecules to the thiol groups of Cys residues inhibits the ability of tau to generate disulfide-linked dimers, which are believed to be the templates for the protein conformation conversion required for tangle assembly [114]. Although the disruptive activity of NE towards tau protofilaments is still elusive, its R3 and R4 regions remain the main targets for the development of therapeutic strategies that aim to clear the neurofibrillary tangles. In tau protofilaments, R3–R4 domains show eight structural β-sheets (β1–β8) that hold up the full length of fibrils; therefore, impairments in those regions result in tau destabilization and degradation. Wan et al. [115] have shown that, due to the binding affinity between NE and aromatic residues (mediated by π–π stacking) and/or negatively charged residues (via hydrogen-bonding interactions), norepinephrine is capable of targeting β-sheets, mostly disrupting β6/β8 and changing the β2–β3 and β6–β7 angles. These alterations on the *C*-shaped tau R3–R4 protofilaments result in reduced aggregate formation; based on these results, NE and other catechol derivatives have attracted great attention as therapeutic anti-aggregation agents, and are not only confined to AD.

### 2.3. NE and α-Synuclein Protein in PD

**Figure 6:**
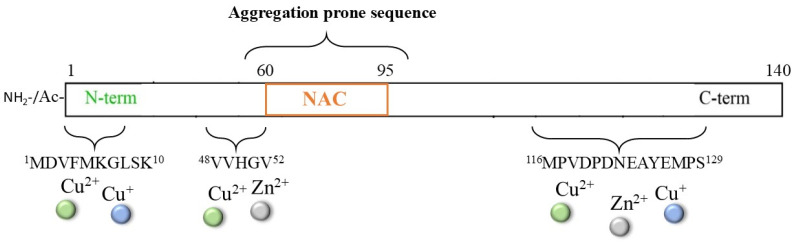
Scheme of the αS protein and of the principal sequences involved in metal interaction.

The multifactorial pathogenesis of Parkinson’s disease (PD) is widely associated with the toxic aggregation of α-synuclein (αS), a pre-synaptic protein required for the recycling of neurotransmitter vesicles and the maintenance of catecholamines levels [116,117,118,119,120]. αS is a small cytosolic protein (~14.5 kDa) that is intrinsically unstructured and presents a NAC core (61–95), which is highly hydrophobic and fibrillogenic; it also presents a *C*-terminal domain (96–140) with a high content of negatively charged residues that are involved in the modulation of filament assembly (Figure 6) [121].

Although a non-specific metal binding site, particularly for iron ions, can be provided by the DPDNEA sequence in the αS *C*-terminus, the *N*-terminal domain 1–60 is commonly accepted as the main anchoring site for transition metal ions (including Zn^2+^, Cu^2+/+^, and, partially, Fe^3+/2+^) due to the presence of His50. The correlation between the generation of metal/αS species and protein aggregation has been extensively reviewed [122,123,124,125,126], together with the physiological implications of metal-mediated post-translational modifications of the αS protein; indeed, new strategies able to target this complex process have been explored. One strategy that has been proposed for inhibiting/disaggregating αS deposits is the use of NE and other catechol derivatives [39,127,128,129,130,131], as previously described for other amyloidogenic-related disorders. Indeed, the presence of high contents of catecholamines (in particular, DA and NE) in dopaminergic neurons indicates that these molecules may be constitutive in the modulation of αS aggregation. Moreover, if the application of NE on Aβ aggregates led to some controversies due to the strong interaction between NE and Tyr10 (which could perturb the copper ion coordination sphere and ROS formation) [85], in the case of αS protein, the lack of tyrosine residues could simplify the mechanism of action of NE, making the NE/αS interaction totally independent of metal/αS binding.

However, when Fisher et al. [130] analyzed the αS fibrillation process in the presence of NE, a higher accumulation of small oligomeric structures that exhibit higher toxicity was observed. Indeed, NE showed the ability to inhibit the generation of large αS fibrils and to disaggregate the preformed deposits, and the stabilization of the resulting small soluble oligomers appeared to be dependent on the extent (see below) of the oxidation of the neurotransmitter. At a neutral to basic pH, NE follows a DA-like oxidation pathway, which results in leuconoradrenochrome, followed by noredrenochrome and noradrenolutin; however, the rate of this process is significantly slower than that of dopamine [132]. Since the generation of melanic structures requires a longer time compared to DA, NE and its soluble oxidation products may be more effective at preserving αS in the monomeric form or at stabilizing the oligomers. On the other hand, since the αS protein does not have highly nucleophilic cysteine residues and the lysine residues are largely protonated, αS/NE covalent modification seems to be unlikely. Singh et al. [133] performed an accurate analysis of the morphological changes in αS species managed by the presence of NE, and observed a weak binding affinity for both initial and intermediate species of αS fibrillation, leading to small and highly toxic annular protofilaments with a high β-sheet content. Moreover, they proposed that the interaction of NE with αS may occur in the NAC (mainly in ^70^VVTGV^74^ and ^80^KTVEG^84^) and the *C*-terminal (^94^FVKKDQLG^101^) regions by establishing specific *H*-bonds and hydrophobic interactions; the binding with these two domains, which are critical for αS fibrillation, would impart a physical block in αS assembly, triggering the formation of highly ordered and kinetically stabilized oligomers. The electrostatic/hydrophobic nature of the interaction between NE/αS was also confirmed by Zou et al. [134], although the proposed binding site was relocated in the Greek-key-like core (44–96) of the protein. The Greek-key-like core in the αS protein consists of the minimal sequence able to generate the smallest aggregates that are thermodynamically stable in solution and able to promote fibril growth [135,136]; the sequence (44–96) is indeed capable of giving rise to protofibrillar trimers, which are the minimal nuclei needed for fibrillation, and tetramers, which are the smallest stable species during the aggregation pathway. Although the smaller size of dopamine, if compared to NE, enhances the accessibility of the catecholamine to the protofibril core, when NE molecules are present in solution, they significantly destabilize the β-sheet structures and the inter-chain E46-K80 salt bridges, thus inhibiting the in vitro formation of αS fibrils and partially dissolving the pre-existing assemblies.

The complexity of this scenario is increased by the presence of NE metabolites and their direct/indirect effect on αS. It is possible to hypothesize that the DOPEGAL-mediated activation of AEP in dopaminergic neurons produces cytotoxic effects; indeed, previous studies have suggested that active AEP cleaves αS at Asn103, resulting in cleavage products that are highly prone to aggregation. This results in neuronal degeneration in the SN [137] and motor dysfunction in PD animal models [104].

Therefore, NE and derivatives play critical and discordant roles in amyloidogenic disorders; they are crucial for AEP upregulation and are able to module the deposition of the aggregates and the release of soluble toxic species of Aβ, αS and tau. Their fibrillar forms may act as growth centers in the initiating events of the neuromelanin biosynthetic pathways, attracting oxidative products of NE, together with metal ions and lipids; a more detailed description of this pathway is provided in the next section.

## 3. Metal-Catalyzed Oxidation and Polymerization of NE Leading to Neuromelanic Species

An important aspect of AD and PD is the loss of noradrenergic neurons in the *Locus coeruleus* (LC) [138], which are the neurons responsible for the synthesis of the neurotransmitter NE [139]. As stated in the first chapter, the degeneration of LC neurons is linked to several motor and non-motor symptoms, including cognitive decline, mood disturbances (e.g., depression and anxiety), and autonomic dysfunction [140]. In pathological conditions, neuronal loss in the LC with a reduction in NE levels worsens the neuroinflammatory and oxidative stress responses in the brain, accelerating the degeneration of dopaminergic neurons in the SN [141].

**Figure 7:**
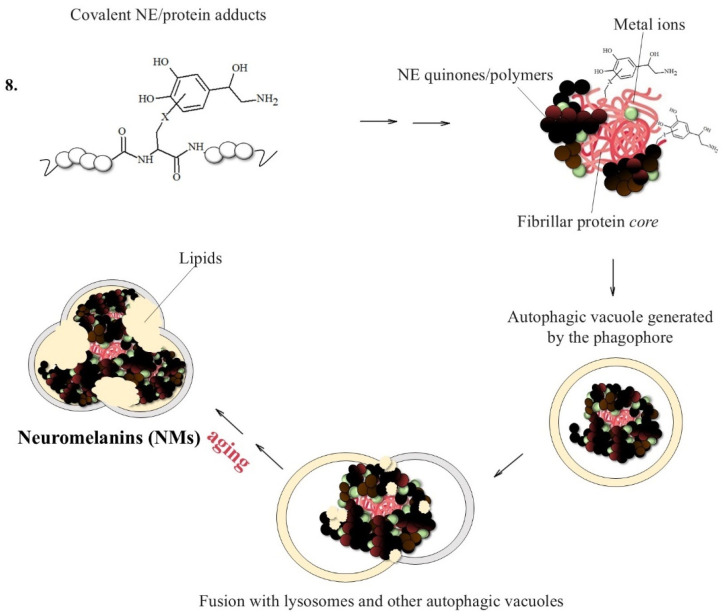
NM formation promoted by the presence of fibrillar protein seeds able to quickly react with NE quinones. NMs initially trap free metal ions, particularly Fe and Cu ions; these structures are then converted into cytoplasmic autophagic vacuoles which are fused with lysosomes and lipid components, forming large organelles surrounded by a double membrane that persist in neurons until cellular death.

As with DA in the *Substantia nigra*, the small amounts of NE leaking from the specialized cytoplasmic vesicles that store the neurotransmitter evolve through a complex biosynthetic pathway that initially involves the oxidation of this catecholamine and ends in the formation of NMs (Figure 7) [138].

The relevance of norepinephrine-containing NMs to neurodegeneration is suggested by the observation that neuropathological studies and Magnetic Resonance Imaging of NMs have shown that, in AD, the first cells to degenerate are the noradrenergic neurons containing NMs of *Locus coeruleus* [15,141,142].

NMs are dark pigments composed of melanic, lipid, and peptide moieties linked together with covalent bonds; also, they bind metal ions, particularly iron, copper and zinc. It is essential to underline that redox metal ions, in particular iron and copper, probably catalyze the initial steps of NM biosynthesis [143,144], promoting the oxidation of catechols to quinones, which react with the cysteine, histidine and lysine of the fibrillar protein seeds.

Once the adducts between the protein aggregates, iron and copper have been formed, they are incorporated by the double membrane to form an autophagosome, which fuses with the lysosome to produce an autolysosome since they are not easily degradable by the proteasome system. NMs will only be obtained when the formed autolysosome fuses with other vesicles containing additional proteins and lipids, a process that continues as the neuron ages [42,145,146,147].

Indeed, the formation of NMs appears to have a protective role. Its biosynthesis removes excess cytosolic catechols, preventing their oxidation into reactive toxic quinones. Additionally, it can sequester the reactive quinones and some xenobiotics involved in neurotoxicity [148].

Besides the Fe, Cu, and Zn ions mentioned before, NMs can chelate, with high affinity, several metals deriving also from environmental exposure (including Al, Cr, Mo, Pb, and Hg).

By binding the potentially harmful iron and copper metal ions, NMs prevent them from activating redox reactions that could otherwise lead to ROS production [5]. This protective function is particularly important in LC and SN, where high levels of metabolic activity and catecholamines can increase oxidative stress.

In NMs, iron(III) can be present in low and high-affinity sites. In the former, iron(III) is present in mononuclear complexes with a distorted octahedral arrangement, coordinated by the oxygen atoms from the catechol portions; in addition, it has a high-spin configuration, detectable by EPR spectroscopy. In high-affinity sites, however, iron(III) is present in ferritin-like clusters connected by oxo and hydroxo bridges, and is undetectable by EPR [141,142]. The two iron coordination modes influence its reactivity; in the latter form, iron is almost redox inactive, while in the mononuclear species, it retains some redox reactivity. This means that the chelation of metals by NMs greatly reduces their ability to generate reactive species, but without eliminating it.

If NM-containing neurons die, there is a huge release in the extracellular space of NMs that causes the strong activation of microglia, leading to a neuroinflammatory cascade. The reactive species and proinflammatory molecules released by microglia are harmful to neighboring neurons, leading to their death and the subsequent discharge of more NMs. During the degradation of extracellular NMs, all toxic molecules accumulated over many years of aging are released and can exacerbate these processes [45,149].

However, a detailed characterization of the NM structure is hampered by the fact that NMs are generally insoluble and can only be extracted in small amounts from the human brain (typically 1.0 mg of NMs from SN requires 4–5 brains of suitable subjects, which reduces to about 1/10 for NMs from LC) [143]. Therefore, in order to better understand the structure and the reactivity of NMs, and particularly those of norepinephrine-containing NMs, synthetic NMs were developed by our group by using different fibrillar proteins as a central nucleus that was highly accessible for a covalent reaction with DA or NE quinones, thus promoting melanin growth [150,151,152].

## 4. Conclusions

An interesting scenario that emerges from previous chapters is that protein modification assumes a highly relevant role in the progression of these neurodisorders, in which uncontrolled levels of metal ions and cytosolic catecholamines, including NE and its oxidative products (principally the quinone species), become crucial in promoting protein damage.

Covalent modifications of the most relevant neuronal proteins and peptides may alter their physiological roles as well as their structural stability, possibly resulting in different tendencies regarding the formation of insoluble deposits. This type of modification is also able to block protein degradation by the chaperone mediated autophagy (CMA) system [153], thus inducing a self-sustained process of protein modification and fibrillation. Protein aggregates may participate in physiological or pathological processes depending on the cellular context: they can (*i*) remove toxic peptide monomers and oligomers, excess cytosolic metal ions, and circulating catecholamines from brain cells, thus acting as neuroprotective agents, or they can (*ii*) activate inflammatory responses as well as the generation of oxygen radicals to aggravate neurotoxicity processes.

Moreover, the presence of β-folded protein centers represents the perfect precursor nucleus required for building NMs, where new molecules of NE quinones can increase the size of the particles by reacting with the highly accessible nucleophilic groups (mainly Cys and Lys) present in β-sheet proteins. Consequently, the presence of NE/protein conjugates as well as their related metal/ROS-dependent pathways plays a fundamental role in PD and AD pathogenesis, governing chronic inflammation, cellular vulnerability to oxidative stress, and neuronal degeneration.

Currently, several therapeutic strategies that aim to contrast proteotoxicity by targeting protein deposition (i.e., by inhibiting or disaggregating preformed aggregates) or by modulating protein expression (in terms of regulation of gene expression or of stimuli responses) have been developed [154,155,156,157]. On the other hand, the deposition of proteins like αSyn, tau and Aβ also appears as a compensatory and protective pathway that should not be chosen as a therapeutic target [43]. Similarly, the inhibition of NM synthesis is often associated with the rapid progression of these neurodisorders. Different chelating agents have been also applied in AD and PD to adjust the overload of metal accumulation [158,159,160], particularly when stored inside NMs, thus preventing metal-induced toxicity after the degradation of NM organelles.

On the other hand, the pathways involved in NE-based NM formation and NE-mediated protein covalent modification are only partially understood; the study of synthetic NM models could provide a powerful tool for elucidating the synthetic, structural and functional features of the protein/NE conjugates. Furthermore, these synthetic preparations are able to mimic the activation of the complex pathways usually associated with neuronal degeneration, such as microglia activation and the chronic neuroinflammatory response [161,162,163].

Neuroprotein deposits and NMs could be used as in vivo biomarkers to monitor the progression of neurodegenerative disorders; the presence of high levels of metal ions, particularly Cu(II) and Fe(III) ions, in NMs as well as in protein deposits would be detected by MRI techniques, providing a quantitative imaging biomarker that can be used to obtain high-resolution neuronal images for the early diagnosis of AD and PD.

## Figures and Tables

**Table 1:** Most common ROS-mediated modifications of amino acid residues inside peptides and proteins.

(*i*) Oxidation of histidine residue	(*ii*) Oxidation of methionine residue
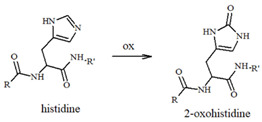	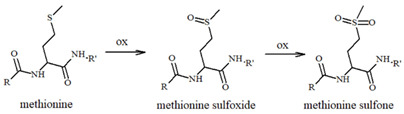
(*iii*) Oxidation of phenilalanine residue	(*iv*) Oxidation of lysine residue
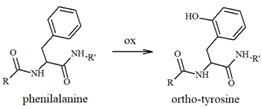	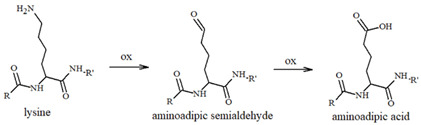
(*v*) Oxidation of tyrosine residue	
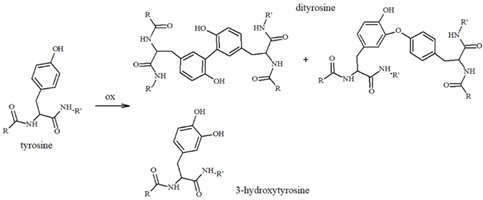	
(*vi*) Oxidation of tryptophan residue	(*vii*) Oxidation of cysteine residue